# Defining the Cell Wall, Cell Cycle and Chromatin Landmarks in the Responses of *Brachypodium distachyon* to Salinity

**DOI:** 10.3390/ijms22020949

**Published:** 2021-01-19

**Authors:** Elzbieta Wolny, Aleksandra Skalska, Agnieszka Braszewska, Luis A. J. Mur, Robert Hasterok

**Affiliations:** 1 Plant Cytogenetics and Molecular Biology Group, Institute of Biology, Biotechnology and Environmental Protection, University of Silesia in Katowice, 40-032 Katowice, Poland; askalska@us.edu.pl (A.S.); agnieszka.braszewska@us.edu.pl (A.B.); 2 Institute of Biological, Environmental and Rural Sciences (IBERS), Aberystwyth University, Aberystwyth SY23 3DA, UK; lum@aber.ac.uk; 3 College of Agronomy, Shanxi Agricultural University, Taigu, Jinzhong 030801, China

**Keywords:** *Brachypodium distachyon*, cell cycle, epigenetics, germination, replication, salt stress

## Abstract

Excess salinity is a major stress that limits crop yields. Here, we used the model grass *Brachypodium distachyon* (Brachypodium) reference line Bd21 in order to define the key molecular events in the responses to salt during germination. Salt was applied either throughout the germination period (“salt stress”) or only after root emergence (“salt shock”). Germination was affected at ≥100 mM and root elongation at ≥75 mM NaCl. The expression of arabinogalactan proteins (AGPs), *FLA1*, *FLA10*, *FLA11*, *AGP20* and *AGP26*, which regulate cell wall expansion (especially *FLA11*), were mostly induced by the “salt stress” but to a lesser extent by “salt shock”. Cytological assessment using two AGP epitopes, JIM8 and JIM13 indicated that “salt stress” increases the fluorescence signals in rhizodermal and exodermal cell wall. Cell division was suppressed at >75 mM NaCl. The cell cycle genes (*CDKB1, CDKB2, CYCA3, CYCB1, WEE1*) were induced by “salt stress” in a concentration-dependent manner but not *CDKA*, *CYCA* and *CYCLIN-D4-1-RELATED*. Under “salt shock”, the cell cycle genes were optimally expressed at 100 mM NaCl. These changes were consistent with the cell cycle arrest, possibly at the G1 phase. The salt-induced genomic damage was linked with the oxidative events via an increased glutathione accumulation. Histone acetylation and methylation and DNA methylation were visualized by immunofluorescence. Histone H4 acetylation at lysine 5 increased strongly whereas DNA methylation decreased with the application of salt. Taken together, we suggest that salt-induced oxidative stress causes genomic damage but that it also has epigenetic effects, which might modulate the cell cycle and AGP expression gene. Based on these landmarks, we aim to encourage functional genomics studies on the responses of Brachypodium to salt.

## 1. Introduction

Throughout their life cycles, plants are exposed to a range of environmental stresses, including salinity, flooding, heat, drought and cold. The prevalence of these stresses has been greatly increased by human activities [1]. Currently, a high level of salinity is among the most important abiotic stresses that reduces crop productivity. Soil salinity is harmful to agriculture because of the effects of osmotic stress and ion toxicity on the growth and development of crop plants. The toxic effects of increased salinization include damage to the cell organelles, the plasma membrane, the disruption of respiration, photosynthesis, protein synthesis and the disruption of the structure of enzymes [2]. An altered water status also results in a decrease in growth by inhibiting cell division, expansion as well as unscheduled cell death. As a result, plants have to adjust the physiological and biochemical processes that regulate ion and osmotic homeostasis as well as the control and repair of stress damage. High salinity also induces ionic and oxidative stress [3], the latter of which is reflected by an imbalance between the generation of reactive oxygen species (ROS) such as the hydroxyl radicals, superoxide and hydrogen peroxide, and ROS scavenging. An excess accumulation of ROS causes oxidative damage to the cellular components such as the membrane lipids, enzymes and nucleic acids, which leads to cell death [4].

Although salt stress affects all of the growth stages, seed germination and seedling growth are the most sensitive phases. After germination, successful seedling formation requires the activation of the cell cycle. The cell cycle progression is driven by the cyclin-dependent kinases (CDKs), which cooperate with the cyclins. There are seven groups of CDKs (CDKA to CDKG) and seven classes of cyclins (CYC) in plants. In plants, the A, B and D cyclin classes have been defined [5]. The A-type cyclins are expressed during the S, G2 and M phases, while the B-type cyclins accumulate during the G2-M phases and regulate the entry into the M phase. The D-type cyclins control the progression from the G1 to S phase in response to external growth signals, but some of them are also involved in the G2 to M transition [6]. Stress negatively affects plant growth and the decrease in plant size can be attributed to a decrease in the number of cells. The latter arises from the inhibition of the CDK activity, which results in cell cycle arrest at the G1/S and G2/M checkpoints, a prolonged S-phase progression or a delayed entry into mitosis [7]. Both the biotic and abiotic stresses that affect plants can influence the cell cycle progression. It was shown that temperature stress arrests the cell cycle due to the reduced transcription of *CYCA* and *CYCB* and, as a result, decreased CDK activity [5]. In wheat seedlings that had been subjected to mild water stress, the mitotic activity was reduced in the mesophyll cells due to the decreased activity of *CDKA1*, which is required for entry into mitosis [5,8].

Plant growth depends on coordination between the mitotic cell cycle and cell expansion; the latter is strongly influenced by the cell wall characteristics. The primary cell wall is typically composed of cellulose, pectins, hemicelluloses and proteins and proteoglycans and glycoproteins are abundantly represented among them. The hydroxyproline-rich glycoprotein superfamily comprises three main families, arabinogalactan proteins (AGPs), extensins and proline-rich proteins [9]. Arabinogalactan proteins are heavily glycosylated proteoglycans that belong to the hydroxyproline-rich glycoprotein superfamily. They influence processes such as cell differentiation, division, tissue development and defense [9,10] by modulating cell wall expansion [9]. Based on the protein structure and polypeptide core, the AGP family is divided into six subfamilies. One of them is the fasciclin-like AGPs (FLAs) subfamily [11]. FLA proteins contain one or two AGP-like glycosylated regions and one or two fasciclin-like (FAS1) domains [12,13,14]. The number of AGP and FAS domains might determine the role of each FLA [15]. To date, *FLAs* have been identified in several plant genomes such as Arabidopsis (*Arabidopsis thaliana*), rice (*Oryza sativa*), wheat (*Triticum aestivum*), cotton (*Gossypium hirsutum*), Chinese cabbage (*Brassica rapa*), eucalyptus (*Eucalyptus grandis*), poplar (*Populus trichocarpa*) and textile hemp (*Cannabis sativa*) [16,17,18,19,20,21]. Despite the importance of AGPs in the abiotic stress responses and plant development [22,23], little is known about their function during plant germination under the stressful conditions in which a disturbance of the cell cycle is an additional limiting factor. In *B. distachyon* (Brachypodium), AGPs have been shown to be involved in temperature stress [24], but they have yet to be investigated in the context of the response to salt stress.

Epigenetic control is also intimately associated with regulating the cell cycle with for example, the phosphorylation, methylation and acetylation of histones that occur in different phases of the cell cycle. However, unlike transcriptional changes, post-translational changes occur throughout the cell cycle. Further, the cell cycle and epigenetic changes are intimately associated with the plant stress response [25,26]. The activation of these stress pathways results in the transcriptional reprogramming and regulation of the genes that are involved in diverse epigenetic processes such as DNA methylation, histone modifications, chromatin remodeling and modulating the expression of non-coding RNA [27]. The effect these have on chromatin have been shown to affect the gene expression under drought, salinity, heat and cold stress in plants [28,29]. As an important epigenetic process, DNA methylation can regulate the plant responses to environmental stresses such as salinity and heavy metal stress in wheat and barley [30,31,32]. A decrease in the global DNA methylation level was observed in two bread wheat cultivars (the salinity-tolerant wheat cultivar SR3 and the salinity-susceptible wheat cultivar JN177) after exposure to increased salination [27,32]. Salt can result in the hyper- or hypomethylation of DNA and these changes are genotype-, tissue- and developmental stage-dependent [33]. Such epigenetic changes are also likely to be influenced by the ROS metabolism during plant growth as well as environmental acclimation [34]. Other epigenetic changes were observed on the histones (H2A, H2B, H3, H4 and the linker H1) and involve the modification of key lysine (K) residues. Histone methylation/demethylation and acetylation/deacetylation are known to regulate many biological processes in plants including the responses to biotic and abiotic stresses [27]. For example, drought, heat and salt stress increases the acetylation of histone H3 at the lysine 9 (H3K9ac) and histone H4 at the lysine 5 (H4K5) sites in *Zea mays* tissues, whereas in rice seedlings, drought also affects the acetylation of histone H3 at lysine 18 and 27 (H3K18ac and H3K27ac, respectively). In Arabidopsis, drought stress leads to changes in H3K9ac and in histone H3 dimethylation and trimethylation at lysine 4 (H3K4me2 and H3K4me3) [33].

Cell cycle and epigenetic effects under stress have been extensively characterized in models such as Arabidopsis and also in cereal crop species but these studies may be considered to be limited. In particular, with crops the domestication process and genetic bottlenecking, could have led to changes which do not fully reflect the effects of natural selection on the genome. Therefore, the exploitation of the genetic resources that are represented by the wild relatives is a promising approach for finding interesting genetic traits than could be translated into improving the breeding strategies of crops [35]. Brachypodium is a close wild relative of many temperate cereals and forage grasses. As a well-established model plant, it has been included into fundamental research on, e.g., plant development, plant-microbe interactions, abiotic stress, evolutionary biology, ecology research as well as for developing new tools aimed at improving other temperate C_3_ cereal crops such as wheat and barley [36].

Investigation of salt tolerance in plants requires morphological, physiological, cellular as well as molecular analyses. Shavrukov [37], highlighted how treatment with salinity may be performed in two forms: as “salt stress” or “salt shock”. During the former a plant is exposed to gradual exposure of increasing salt levels, whereas the latter consists of exposing a plant suddenly to high levels of salinity. Both “salt stress” and “salt shock” consist of osmotic and ionic component with resulting different patterns of gene expression [37]. In *Beta vulgaris* “salt shock” elicits greater transcriptomic changes than “salt stress” and it results in greater number of up-regulated genes compared to the latter [38]. As only a few studies examined the effects of “salt stress” and “salt shock” in parallel experiments, we performed for the first-time analyses of the role of salinity during Brachypodium seed gemination and seedling development. Herein, we use Brachypodium to determine the impact of excess salt (NaCl) on the key cell wall genes, the cell cycle and histone modifications.

## 2. Results

### 2.1. Seed Germination, Root Length and the Arabino-Galactan Protein Gene Transcript Profiles

**Figure 1:**
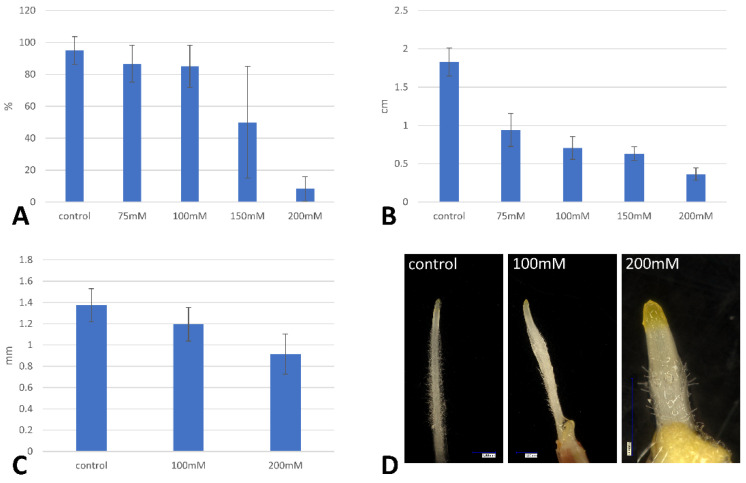
Effect of salinity (various concentrations of NaCl) stress on the percentage of seed germination (**A**), root length (**B**), root tip length (**C**) and root morphology (**D**) in Brachypodium. The results that are shown are the means ± SD from three replicates (*n* = 3).

The inbred Brachypodium accession Bd21 had an appreciable tolerance to NaCl treatments as applying 75 mM and 100 mM did not significantly inhibit the germination of Brachypodium at the “salt stress” conditions. The inhibition was more pronounced at higher concentrations of salt treatment for which the germination rates decreased to 50% of the controls at 150 mM NaCl (Figure 1A). The lowest germination was observed with 200 mM of NaCl where only a single seed of Brachypodium exhibited a radicle protruding through its coleorhiza.

Root length is an important indicator of salt stress tolerance since it is the first organ to come into contact with the soil and therefore with salt. Increasing the NaCl concentrations resulted in a significant decrease in the root length of the Brachypodium seedlings (Figure 1B). An approximately 50% decrease in the average root length was observed at the lowest treatment of NaCl, 75 mM. The degree of root growth inhibition increased with salt concentration and after 200 mM NaCl were only a few millimeters long (Figure 1B). Higher salt concentration influenced not only the length of the whole roots, but also the length of the root tip, which shortened as the salinity increases (Figure 1C,D). In addition, the number and length of root hairs were also affected (Figure 1D).

**Figure 2:**
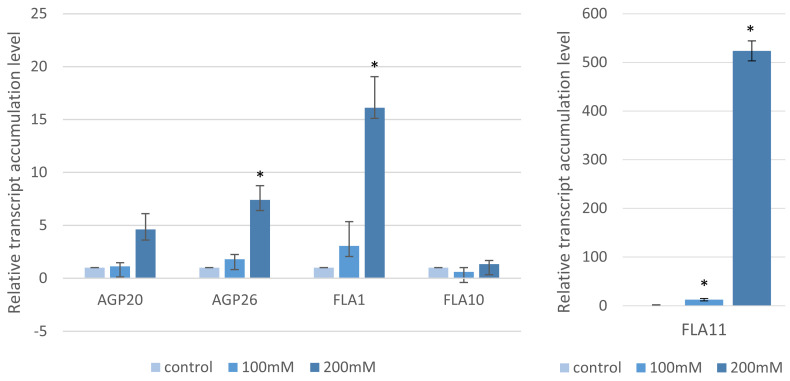
Relative level of the accumulation of the arabinogalactan proteins (AGP) and fasciclin–like AGP (*FLA*) gene transcripts after “salt stress” treatment with various concentrations of NaCl. The relative expression levels were normalized to an internal control (Bradi1g32860; encoding ubiquitin) and calibrated to the control. Asterisks indicate significant differences from the control using the Student’s *t*-test (*p* < 0.05; mean ± SD, *n* = 3).

**Figure 3:**
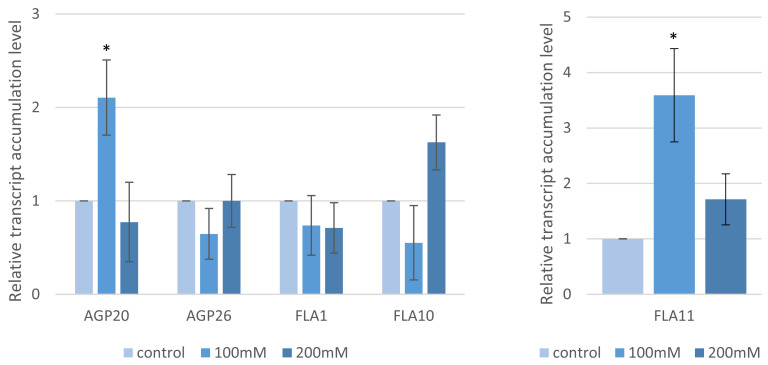
Relative level of the accumulation of the AGP and fasciclin–like AGP (*FLA*) genes after “salt shock” treatment with various concentrations of NaCl. The relative expression levels were normalized to an internal control (Bradi1g32860; encoding ubiquitin) and calibrated to the control. Asterisks indicate significant differences from the control using the Student’s *t*-test (*p* < 0.05; mean ± SD, *n* = 3).

These phenotypic changes were further studied by assessing the expression of the five genes encoding the arabinogalactan proteins (AGPs), which are regulators of cell wall expansion, using RT-qPCR. The following genes were selected: Bradi2g00220*—FLA11,* Bradi5g18950*—FLA10,* Bradi4g34420*—FLA1,* Bradi2g45510—*AGP20* and Bradi2g60270*—AGP26*. Two experimental conditions were assessed: (i) after the application of “salt stress” at germination for a period of three days and (ii) “salt shock”, which was induced by the transfer of germinated seeds into NaCl solutions for 12 h. Under “salt stress” conditions, the transcript accumulation of these genes increased with elevating NaCl concentrations (Figure 2). The expression of Bradi2g00220 (*FLA11*) had the most marked result with a 633.4-fold change at 200 mM NaCl compared to the controls. The differences in Bradi4g34420 (*FLA1*)*,* Bradi2g45510 (*AGP20*) and Bradi2g60270 (*AGP26*) were more modest but still showed statistically significant differences of 16.1-, 4.6- and 7.4-fold, respectively, after the 200mM NaCl treatments compared to the controls. The expression of Bradi5g18950 (*FLA10*) had no significant differences to the controls. When the alternative “salt shock” approach was used, only the expression of Bradi2g45510 (*AGP20*) and Bradi2g00220 (*FLA11*) was significantly different at 100 mM compared to the control (2.1- and 3.5-fold, respectively) (Figure 3).

### 2.2. Distribution of the AGP Epitopes in Roots in Response to Salt Stress

**Figure 4:**
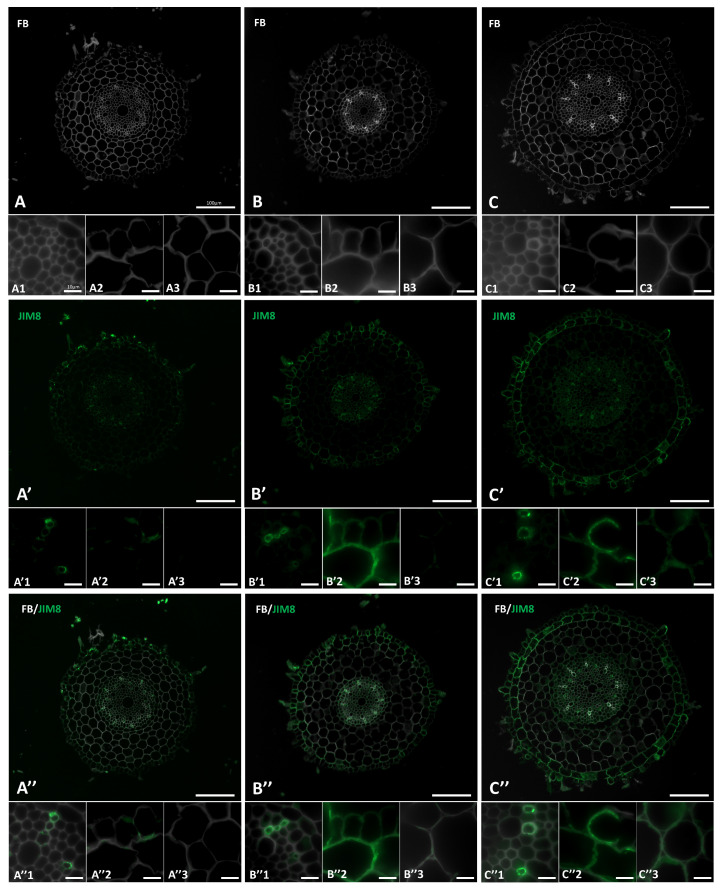
Immunolocalization of the JIM8 epitope in cross-sections of the “salt stressed” Brachypodium roots. (**A**,**A′**,**A″**) control, (**B**,**B′**,**B″**) 100 mM NaCl, (**C**,**C′**,**C″**) 200 mM NaCl. Fragments of vascular cylinder are marked by the numeral 1, rhizodermis and exodermis by the numeral 2, and cortex cells by the numeral 3. FB—fluorescent brightener. The green colour shows epitope occurrence. Scale bars for A,A′,A″, B,B′,B″, C,C′,C″ represent 100 µm and for all other photomicrographs: 10 µm.

**Figure 5:**
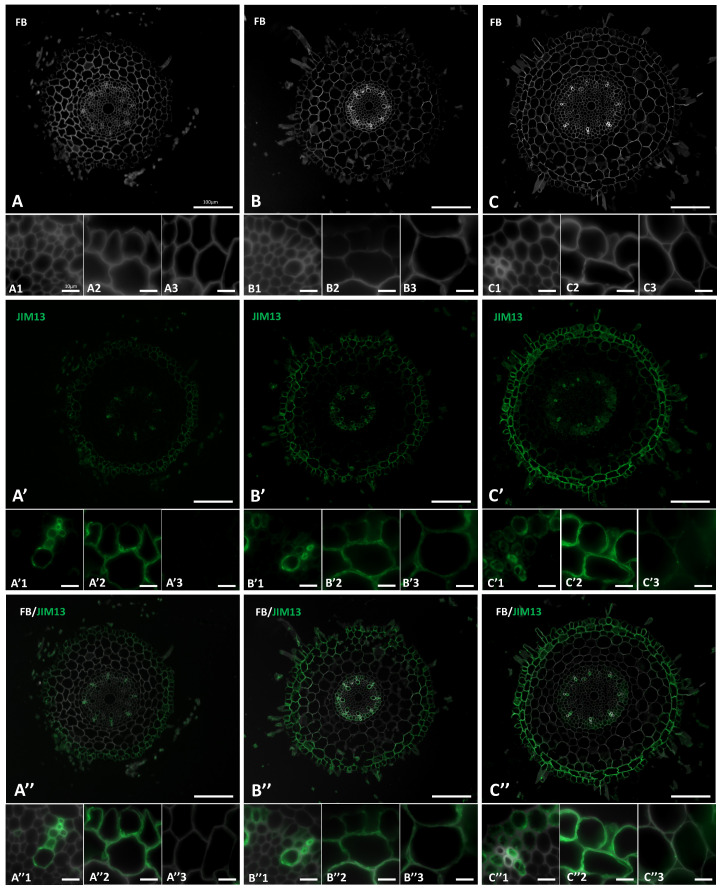
Immunolocalization of the JIM13 epitope in cross-sections of the “salt stressed” Brachypodium roots. (**A,A′,A″**) control, (**B,B′,B″**) 100 mM NaCl, (**C,C′,C″**) 200 mM NaCl. Fragments of vascular cylinder are marked by the numeral 1, rhizodermis and exodermis by the numeral 2, and cortex cells by the numeral 3. FB—fluorescent brightener. The green colour shows epitope occurrence. Scale bars for A,A′,A″, B,B′,B″, C,C′,C″ represent 100 µm and for all other photomicrographs: 10 µm.

The distribution of two AGP epitopes, JIM8 and JIM13 in the roots of Brachypodium under “salt stress” conditions was determined (Figure 4 and Figure 5). Signals from these two epitopes were mostly associated with the vascular tissues, rhizodermis and exodermis. The presence and spatial distribution of the JIM8 epitope was different in the “salt stressed” roots comparing to the control plants. This epitope was less abundant in the control roots (Figure 4A′,A″), but in the roots that have grown under “salt stress” (Figure 4B′,C′,B″,C″) a strong fluorescence signal was observed in walls of rhizodermal and exodermal cells (Figure 4B′2,C′2). In walls of some cortex cells weak fluorescence signals were also visible (Figure 4B″3,C″3). JIM13 was found at the same locations as the JIM8 epitope (Figure 5) but it was additionally detected in the intercellular compartments of the vascular cylinder cells (Figure 5B′1,C′1,B″1,C″1). An increase in the intensity of fluorescence signal of JIM13 was observed in the walls of rhizodermal and exodermal cells (Figure 5B′2,B″2,C′2,C″2) as well as in the walls of cortex cells (Figure 5B′3,C′3,B″3,C″3) in “salt-stressed” roots compared to the control (Figure 5A′3).

### 2.3. The Effect of NaCl on DNA Replication and Mitotic Activity

The inhibitory effects of salt on germination and root growth could imply effects on cell division as well as cell expansion. To assess cell division and the effect of NaCl treatment on the DNA replication in Brachypodium root cells, DNA synthesis was visualized by incorporating EdU into the actively replicating nuclei.

**Figure 6:**
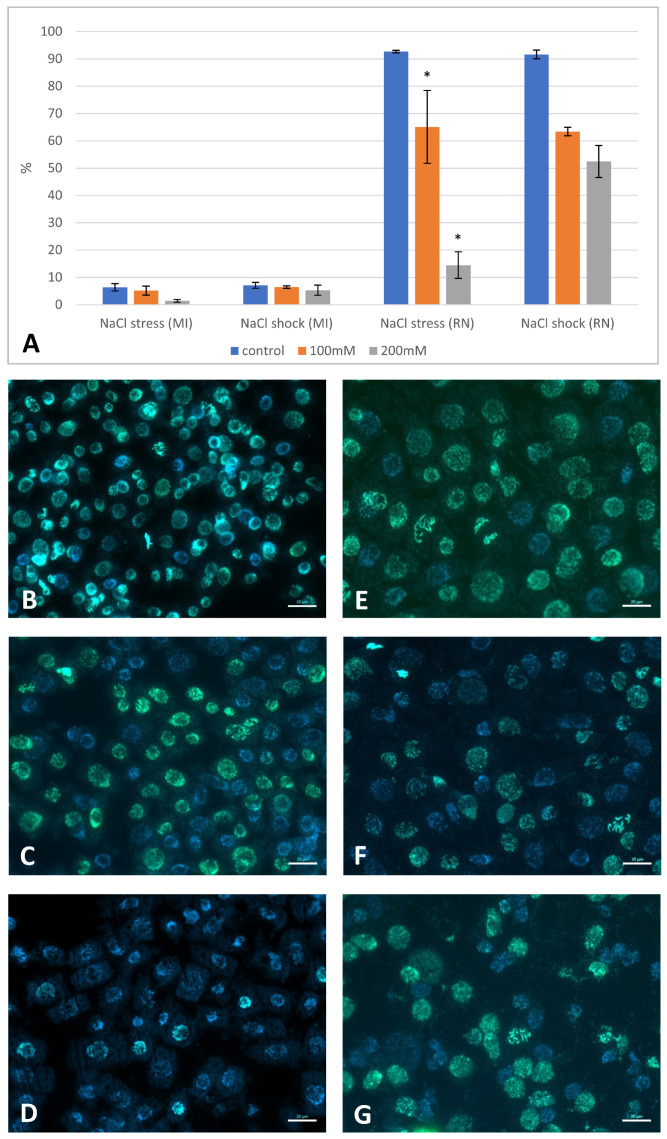
Mitotic activity of the root cells in “salt-stressed” and “salt-shocked” Brachypodium seedlings and the frequency of nuclei with replicated DNA in the root meristematic cells. (**A**) Mitotic index (MI) and the percentage of replicated nuclei (RN) after “salt-stress” and “salt-shock”, which were caused by applying various concentrations of NaCl. Asterisks indicate significant differences from the control; differences between the groups were calculated using the one-way ANOVA (*p* < 0.05; mean ± SD, *n* = 3) with a prior arcsine square root transformation. (**B**–**G**) Detecting the EdU (green fluorescence) that had been incorporated into the actively replicating nuclei in the “salt-stressed” (**B**–**D**) and “salt-shocked” (**E**–**G**) Brachypodium seedlings. (**B**,**E**) control, (**C**,**F**) 100 mM NaCl, (**D**,**G**) 200 mM NaCl. Blue colour—DAPI fluorescence; green colour—Alexa Fluor 488 fluorescence. Scale bars represent 20 µm.

Nuclei at the S-phase or those that had replicated DNA during the final 12 h of growth gave a positive EdU signal, which was visible as a green signal using Alexa Fluor 488. The analysis was performed for both “salt-stressed” and “salt-shocked” Brachypodium seedlings. In both groups, the number of replicating nuclei decreased at higher NaCl concentrations (Figure 6; Supplementary Materials Figure S1). In the control group, although about 90% of the nuclei displayed a positive EdU signal, with 100 mM NaCl salinity, this was significantly reduced with only 65% and 63% “salt-stressed” and “salt-shocked” seedling showing evidence of replication. After applying 200 mM of NaCl to the “salt-shocked” seedlings, the percentage of replicating nuclei was 52%, whereas in the “salt-stressed” seedlings, this decreased to 15% of the nuclei (Figure 6A,D,G). Seed germination in saline conditions also had a negative effect on the mitotic index (MI) in the meristematic root cells, particularly at the higher concentrations (Figure 6A).

### 2.4. Analysis of the Cell Cycle Gene Transcript Profiles

**Figure 7:**
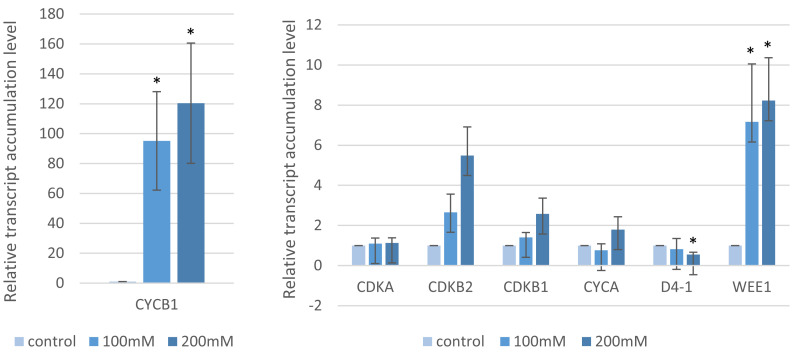
Relative level of the accumulation of the cell cycle gene transcripts after “salt stress” treatment with various concentrations of NaCl. Relative expression levels were normalized to an internal control (Bradi1g32860; encoding ubiquitin) and calibrated to the control. Asterisks indicate significant differences from the control using the Student’s *t*-test (*p* < 0.05; mean ± SD, *n* = 3).

**Figure 8:**
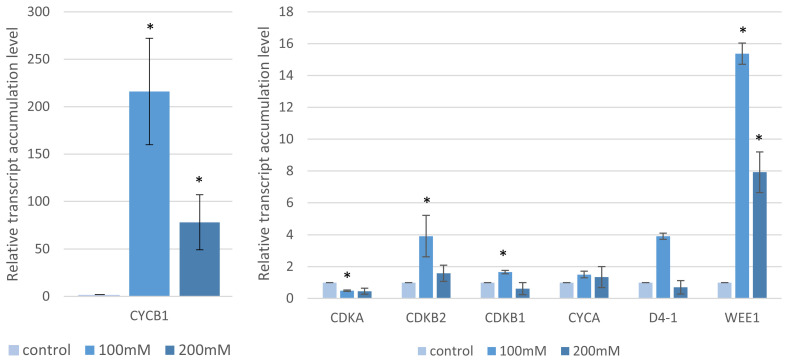
Relative level of the accumulation of the cell cycle gene transcripts after “salt shock” treatment with various concentrations of NaCl. Relative expression levels were normalized to an internal control (Bradi1g32860; encoding ubiquitin) and calibrated to the control. Asterisks indicate significant differences from the control using the Student’s *t*-test (*p* < 0.05; mean ± SD, *n* = 3).

Given the MI result, we sought to describe the impact of salt on the cell cycle by measuring the expression of seven cell cycle-related genes (Bradi3g02270.1—*CDKA,* Bradi4g25980.1—*CDKB1*, Bradi3g40200.1—*CDKB2*, Bradi1g14820.1—*CYCA3*, Bradi2g52760.1—*CYCB1*, Bradi4g32556.1—*CYCLIN-D4-1-RELATED* and Bradi3g03112.3—*WEE1*). Their expression differed depending on both the NaCl concentration (Figure 7) and salt incubation conditions (Figure 8). In the case of “salt stress”, the accumulation of the *CYCB1* transcript increased with an increase of the NaCl concentrations (95- and 120-fold for 100 mM and 200 mM NaCl, respectively). As with *CYCB1*, *WEE1* had a substantial expression. Despite the lack of statistical significance, a similar trend was observed for *CDKB1* (1.4- and 2.5-fold with *p*-value = 0.1 and 0.07, respectively) and *CDKB2* (2.6- and 5.4-fold with *p*-value = 0.33 and 0.056, respectively) after applying 100 mM and 200 mM NaCl. However, no significant changes were observed in *CDKA* and *CYCA* and in the case of *CD4-1,* only minor differences were seen after applying 200 mM NaCl (*p* = 0.02) (Figure 7). However, with “salt shock”, the expression of all of the genes was higher at the 100 mM NaCl solution compared to the controls (Figure 8). The only exception was *CDKA*, which had a lower gene expression level than the control (Figure 8). As with the “salt stress” experiment, there was a highly significant (*p* < 0.001) and dramatic increase in the expression of *CYCB1* (216- and 78.1-fold for 100 and 200 mM NaCl, respectively) and *WEE1* (15.3- and 7.9-fold for 100 and 200 mM NaCl, respectively). Smaller but statistically significant (at least at one concentration) changes in the expression were observed for *CDKB2* (3.9- and 1.5-fold), *CDKB1* (1.6- and 0.6-fold) and *CDKA* (0.49- and 0.45-fold) (Figure 8).

### 2.5. Genomic DNA Damage

**Figure 9:**
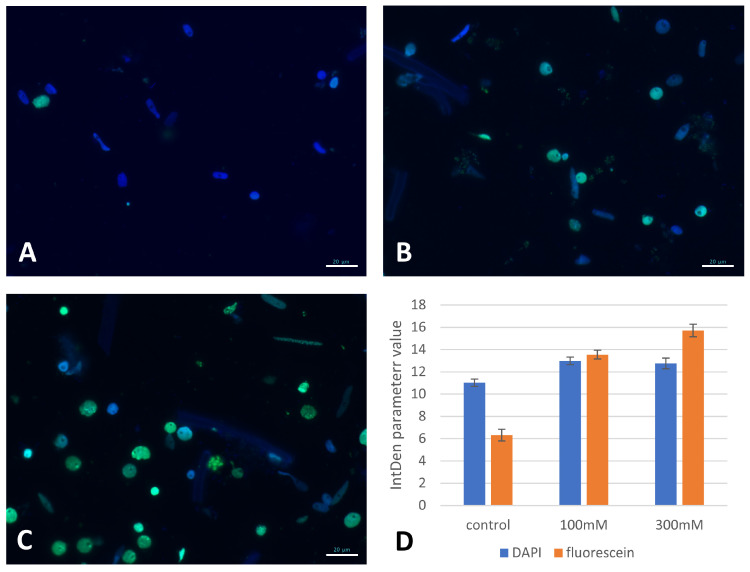
TUNEL test in the root cells of the “salt-shocked” Brachypodium seedlings. (**A**) Control, (**B**) 100 mM NaCl, (**C**) 300 mM NaCl. DAPI stained nuclei with or without green fluorescence of built-in fluorescein as a result of the TUNEL reaction. Levels of relative fluorescence intensity (IntDen parameter) of DAPI and fluorescein (**D**); values are shown as the average values ± SE. Blue colour—DAPI fluorescence; green colour—fluorescein fluorescence. Scale bars represent 20 µm.

TUNEL was used to assess the nuclei for DNA nicks and fragmentation after the salt treatment. TUNEL analyses were performed for the nuclei that originated from the seedling roots that had been subjected to 100 mM and 300 mM “salt-shock”. As most of the nuclei from the NaCl-treated root cells exhibited a green fluorescence (Figure 9B,C), the fluorescein fluorescence was quantified to indicate the degree of DNA damage (Figure 9D). The fluorescein intensity increased with higher NaCl concentrations with the values that were obtained for 100 mM and 300 mM salt > 50% higher than the control.

### 2.6. DNA Methylation and Histone Modifications

**Figure 10:**
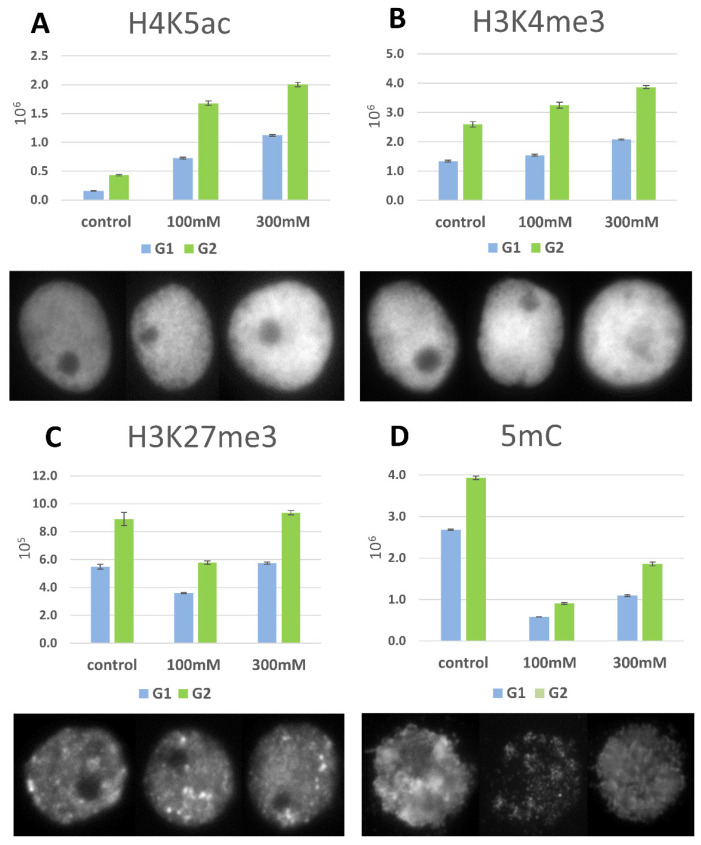
Levels of the relative Alexa 488 fluorescence intensities for the modified histones H4K5ac (**A**), H3K4me3 (**B**), H3K27me3 (**C**) and 5-methylcytosine (5 mC) (**D**) as well as for the immunostaining of representative interphase nuclei in the root cells of the Brachypodium seedlings that had been “salt-shocked” with 100 mM and 300 mM NaCl. The values are shown as the average values ± SE.

DNA methylation and histone H4 acetylation were the most variable epigenetic modifications in the roots of the “salt-shocked” seedlings. The fluorescence signals that were obtained for H4K5ac were four-fold higher in the 100 mM and seven-fold higher in 300 mM “salt-shocked” plants (Figure 10A). The euchromatin-specific modification H3K4me3 was more than one-fold higher in the 100 and 300 mM (Figure 10B) “salt-shocked” plants compared to the controls. By contrast, the heterochromatin-specific modification of H3 trimethylation at lysine 27 (H3K27me3) was more than one-fold lower in the 100 mM salt-treated plants. After the 300 mM NaCl shock, there was an increase in the H3K27me3 fluorescence, but this was less than one-fold higher than for the control plants (Figure 10C). The DNA methylation decreased dramatically after “salt shock” by >four- and two-fold after the application of 100 mM and 300 mM NaCl compared to the controls, respectively (Figure 10D).

## 3. Discussion

Excess salinity is a major factor that limits crop yield throughout the world including the yield of cereals. To provide insights into how grasses and cereal responded to salt, we used the well-established model grass Brachypodium. Our study aims to differentiate between changes occurring during “salt stress” or “salt shock”. The difference could reflect differences between responses to germinating in salty soil as opposed to episodic flooding with salt water as may occur in marine or estuarine environments. The main component of “salt shock” is osmotic stress or plasmolysis, especially in root cells [37,39]. As a consequence, plants exposed to long-lasting “salt shock” can undergo apoptosis-like cell death [40], which was not reported after plant adaptation to the higher presence of NaCl. There are several examples of different gene expressions after salt shock and stress (for review see [37]). Experiments based on “salt shock” lead to the identification of genes which are responsible for defense mechanisms against osmotic shock or plasmolysis [39], but their identification is easier soon after shock induction. On the contrary, experiments with “salt stress” are more likely to lead to the identification of genes which expression is highly connected and important for the reaction and adjustment to stressful conditions.

Based on the precedents in the literature that have been established for Arabidopsis, we sought to link any phenotypic changes to those of the cell cycle and the epigenome. We focused on the seed germination stage (“salt stress”) and three-day old seedlings (“salt shock”) as these are particularly sensitive to the effects of salt stress. Salinity can affect seed germination by creating the osmotic potential to prevent water uptake and/or by providing conditions for the entry of ions that may be toxic to the embryo or developing seedling. At the seed germination stage, salt stress restricts water absorption, inhibits the growth of the embryo axis and leads to secondary seed dormancy [41]. Brachypodium had a decreased germination efficiency under saline conditions. There was a significant decrease in the percentage of germinated seeds at the 100 mM NaCl concentration and at 200 mM, only a few seedlings with a developed radicle were observed. We also observed a significant decrease in the root length in Brachypodium, which indicates that this developmental stage is also sensitive to salinity. The shortness of the root tip length may also indicate disturbances in cell elongation in ”salt stress” conditions. While similar responses to salt stress at the germination stage have also been reported in many crop plants [42], in order to maximize the utility of Brachypodium as a model, we sought to identify some of the underlying molecular events.

Roots respond to salt by modulating the gene expression, metabolism and protein activity, which results in changes in the cell wall composition, transport processes, cell size and shape and root architecture [43]. As an indicator of cell wall-linked events, we investigated the expression of AGPs as they play important roles in salt stress responses. For example, tobacco (*Nicotiana tabacum*) cells that had been adapted to NaCl were characterized by fewer AGP epitopes on the plasma membrane, less cell enlargement and decreased cell wall extensibility [9,44,45]. In agreement with these results, there was a significant downregulation of the AGP, *FLA* genes in rice (*OsFLA10/18*) and wheat (*TaFLA3/4*) under salt stress [16,46]. However, several studies have proven that in the response to adverse environmental conditions, the *FLA* gene expression can be also upregulated. Thus, the Arabidopsis mutant at *fla4* (*SALT OVERLY SENSITIVE 5; SOS5*) was identified as being salt hypersensitive [47]. Further, rice *FLA23*, wheat *FLA12* [16,46] as well as *FLA2/12/21/24/30* genes in *Populus* species were all upregulated under salt stress [20]. In our studies, we observed increases in the *FLA* genes, which were consistent with a salt-responsive modification of the cell walls and concurs with the immunochemistry observations that have been made for the JIM8 and JIM13 antibodies which are specific for AGP. We focused on the expression of the genes encoding *FLA1/10/11* and *AGP20/26* as representatives of the fasciclin-like AGPs, which have two AGP domains and two fasciclin domains. *FLA1* was reported as participating in the early developmental events during root and shoot development [15]*. FLA11* and *FLA12* have been observed to be involved in the secondary cell wall thickening of the stems [48], a co-expression with the secondary cell-wall-specific cellulose synthase loci [49] and expression in sclerenchyma cells [50]. In Arabidopsis, mutations in *AtFLA11* result in altered stem biomechanics with a reduced tensile strength and elasticity as well as altered cell walls that have an increased cellulose microfibril angle, reduced arabinose, galactose and cellulose content and deposition [51]. Structurally, FLA via their FLA1 domains might form a heteromeric higher-order network that strengthens the interaction between the cellulose microfibrils [51]. This being stated, the increased expression of *FLA1/10/11* and *AGP20/26* in Brachypodium did not correlate with, e.g., the decreased root expansion compared to the controls. Further, we were not able to observe any significant changes in the *FLA10* (Bradi4g34420) expression at NaCl concentrations below 200 mM. These results were contrary to those of Guerreiro et al. [17], who showed a significant upregulation of *FLA10* in *Cannabis sativa* at lower concentrations of salt, while the *FLA10* expression was suppressed by salt in two cultivars of *Solanum lycopersicum* [52]. Clearly, *FLA1/10/11* can have very different activities in different plant species. In the case of Brachypodium, this could indicate that salt-induced *FLA1/10/11* could coordinate a reconfiguration of the cell wall as cells shrink and the cell cycle is suppressed. Many authors have demonstrated that in response to abiotic stress, plants overexpress the genes that are involved in cell wall modifications [53,54]. Most surprisingly is the major upregulation of *FLA11* in Brachypodium, although the significance of this is unclear since its expression under excess salinity has not previously been described.

In our research AGP genes alter their expression only in “salt stress” conditions, whereas “salt shock” did not make such effect. AGPs are probably one of the most heterogeneous and complex families of macromolecules, reflecting their diverse biological functions. In particular, much research has shown how different AGP expression can be up- and down-regulated in responses [44,45,55,56,57]. Mareri et al. [58] suggested that AGPs may act as plasticizers under salt conditions, and that the release of different AGPs is important in both stress adaptation and cell–cell signaling. Interestingly, while comparing gene expressions in stress traction length, Zang et al. [20] showed that in *Populus* the expression of different FLAs in root tissues was induced at different time points. This could reflect discrete roles for different FLA in cell–cell communication, cell wall lignification and in the secondary cell wall formation [48,51]. Increased lignification of vascular tissue cell walls was also concluded by Neves et al. [59] as a result of NaCl stress, and the deposition of lignin as an adaptation during salinity stress. However, it is still unclear whether such modifications are either part of the cell damage or cell response [58]. In this study, we used AGP immunolocalization to provide spatial information and this suggested that under “salt stress” increased *FLA* gene expression could be associated with cell wall modifications. Interestingly, expression of the AGP family was not affected by “salt shock”.

**Figure 11:**
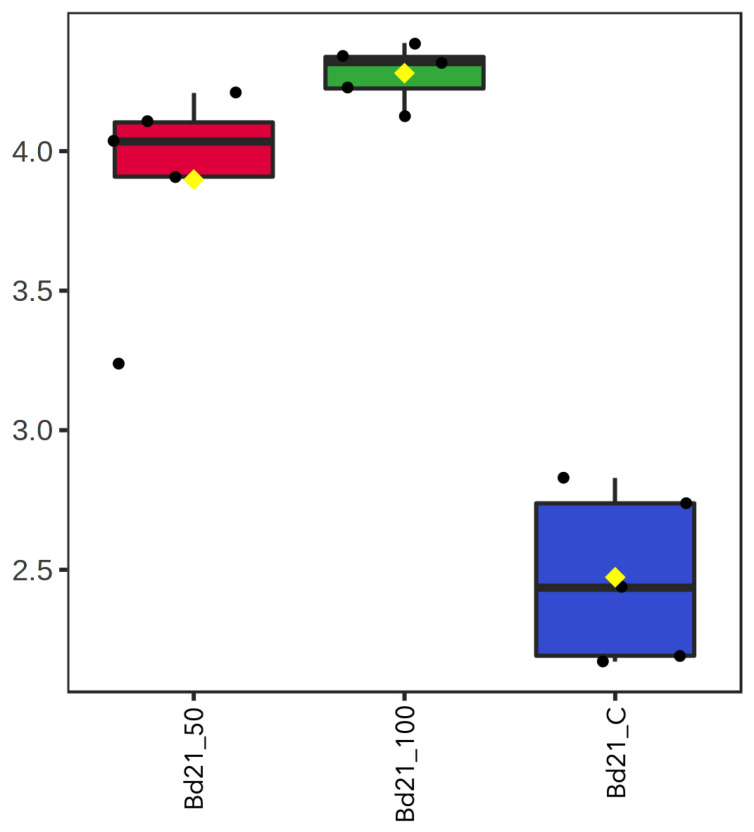
Box and whisker plot of the relative glutathione levels within the Brachypodium Bd21 samples under salt stress conditions. Labels on the *x*-axis refer to the NaCl concentrations (50 mM, 100 mM and the control, respectively). The *y*-axis represents the glutathione content as a % of the total ion count in a given sample. Black dots represent repetitions and yellow diamonds indicate the mean values.

In our study, the presence of salt during germination negatively influenced not only whole root growth but also root tip length which can be correlated with disturbances in cell elongation as well as negative salt affection of mitotic activity and cell cycle progression. The last two were clearly indicated by the suppressed mitotic activity and cell cycle progression during germination under excess salt conditions. This was further indicated by our measurements of the thymidine analogue EdU in the DNA during the S phase of the cell cycle. We used a 12 h EdU labelling to estimate the number of actively replicating cells as this period corresponds to the cell cycle length in Brachypodium. These results clearly indicated a decrease in the frequency of the replicating/ed nuclei in salinity conditions. We then extended this assessment by analyzing the expression of the key cell cycle genes using RT-qPCR. The progression through the different phases of the cell cycle is controlled by CDKs and cyclins, which are their regulatory subunits [6]. Our results indicated that major changes in the transcript levels of the *CDK* and *CYC* genes occurred during “salt stress” and “salt shock”. While “salt stress” had no discernible effect on the *CDKA* gene expression, “salt shock” suppressed its expression, which is consistent with the cell cycle progression being blocked. Additionally, salinity reduced the *CYCD4-1* expression, which would contribute to an arrest of the G1 phase. Another regulatory step is represented by the formation of the CDKB2 and B-type cyclins complexes [60]. CDKBs are the major mitotic regulators in Arabidopsis [61], in which they are degraded and transcriptionally downregulated during DNA damage [62]. Nevertheless, other than in Arabidopsis, only one representative of the CDKB2 group, CDKB2;1, has been shown to accumulate during DNA damage in rice [63]. Here, Brachypodium also showed such an effect with an increased *CDKB2* expression under “salt stress” and “salt shock” conditions. In rice, the higher expression of *CDKB2* is linked with the generation of ROS, and we suggest that this also occurs in salt-stressed Brachypodium. This was indicated by increases of the antioxidant glutathione (Figure 11) and the effects of oxidative stress as likely being caused by the genomic damage, as shown with the TUNEL test. The protective effects of glutathione involve metal sequestration and the scavenging of ROS in plants that are facing environmental stresses [64]. In yeast and mammals, DNA damage activates the ATM (ataxia telangiectasia-mutated) and ATR (Rad3-related) signaling cascades, which simultaneously switch the DNA repair complexes on and arrest cell division [65]. In plants, De Shutter et al. [66] showed that the cell cycle regulatory kinase *WEE1* gene is transcriptionally activated after DNA damage in an ATM-ATR-dependent manner. *WEE1* may therefore be the critical target of cell cycle arrest after the activation of the DNA quality checkpoints. This would act as a link between mitosis and DNA repair [66]. This hypothesis is supported by our results, where the significant increase of the *WEE1* transcript accumulation under both salt stress and salt shock conditions linked the aforementioned CDK effects of salt in Brachypodium and the activation of the DNA repair mechanisms.

The final component of cellular control that we considered in the context of salt effects was its impact on the epigenome. Histone modifications play a crucial role in the plant response to abiotic stresses in order to modulate the developmental cell fate decision via chromatin remodeling and gene silencing [67]. Histone H3 and H4 lysine acetylation is mostly increased during the plant response to stress. Recent results have indicated that in rice plants after submergence or starvation, the genome-wide acetylation of H3K9 undergoes dynamic changes that correlate with the stress-induced gene activation [68]. Our results showed that the total acetylation levels of H4K5 in Brachypodium were higher after treatment with NaCl. This form of histone acetylation promotes open chromatin and gene activation, and therefore the elevated level of H4K5ac under excess salinity conditions may be linked with the higher expression of the cell cycle genes. Zhao et al. [69] found that the abiotic stresses in maize result in histone acetylation changes around the promoter regions of the cell cycle genes that contribute to the control of their expression. Histone modification could also be linked to the cell wall modifications that are implied from our assessments of the AGP expression. In tobacco. BY2 and Arabidopsis T87 cells, high salinity and cold stress triggered the rapid upregulation of histone H3 serine 10 phosphorylation (H3Ser10ph) and histone H4 acetylation, which correlated with the activation of the stress responsive genes. Additionally, in maize roots under salt stress, the upregulation of the cell wall-regulated genes has been associated with the increased acetylation of H3K9 [26,70].

Considering histone methylation, the total levels of H3K4 and 27 trimethylation in Brachypodium under stress were not significantly altered. However, a more detailed whole-genome CHiP-seq study of H3K27me3 indicated methylation changes during the response to salt stress in soybean, which correlated with the activation or inactivation of the salt-inducible genes [71]. Comparative analyses of salt-tolerant and sensitive rice varieties revealed a significant variation in H3K27me3 and H3K4me3, which was linked to the differential expression of the salt-responsive gene OsBZ8 [72]. In castor bean, the key salt-response regulator RSM1, which is a MYB-related transcription factor that is involved in abscisic acid-mediated salt stress signaling, was likely to be regulated through the modification of H3K4me3 and H3K27me3 [73]. Interestingly, our studies suggested an inverse relationship between an increase in the acetylation levels of histone H4 and a significant decrease in DNA methylation. Global changes in DNA methylation following abiotic stress have been reported in several plant species [25]. For example, a decrease in the global DNA methylation levels was observed in bread wheat cultivars after exposure to salinity stress [27,32]. Further, the responses to metal-stress [27,30,74] or the defense response to pathogens in barley and wheat appear to be linked with DNA hypomethylation [27].

A key regulator that influences the epigenetic patterns could be ROS and other redox intermediates. These regulate the activity of many of the enzymes that are involved in DNA methylation, histone methylation and acetylation, which consequently control plant growth, development and the responses to diverse environmental stresses [75]. Increases in ROS result in various histone modifications such as H3K4me2/3, H3K79me3, H3k27me3 and H3K9me2 due to the inhibition of histone demethylases [34]. Redox intermediates not only govern histone modifications but also DNA methylation, where elevated ROS accumulation leads to DNA hypomethylation [75]. Although we did not directly measure ROS in our experiments, oxidative changes are implied from our detection of the increased levels of glutathione (Figure 11). Further, increases in ROS after salt treatments are well supported by the literature [76]. The increased production and accumulation of free radicals during stress can pose a threat to cells at both the structural and genome level by introducing a number of different DNA lesions [34] that can trigger cell death [77]. Given the level of DNA damage in Brachypodium that was detected by the TUNEL test, this is likely to be an effect of ROS.

Taking all of our observations together, it seems likely that salt causes ROS generation in order to alter the influence of histone acetylation and cause DNA hypomethylation. These would affect gene expression, possibly including the genes that lead to cell cycle arrest and AGP expression, which would influence cell wall expansion. These would, in large part, explain the phenotypic effects on germination and root elongation that was observed in Brachypodium following various salt treatments.

## 4. Materials and Methods

### 4.1. Plant Material and NaCl Treatment

Seeds of Brachypodium Bd21 were used in each experiment. Two strategies of NaCl treatment were used in this study. In the first one, the Brachypodium seeds were germinated on a NaCl solution (“salt stress”). The seeds were placed in Petri dishes on three layers of filter paper that had been soaked with distilled water and 10 mL of an NaCl solution (either 0 mM (control), 75, 100, 150 or 200 mM NaCl) (POCH Basic, Gliwice, Poland). After three days, the seedlings were fixed. In the second experimental approach (“salt shock”), unstressed three-day-old seedlings were transferred to a Petri dish with filter paper that had been soaked with 100, 200 and 300 mM NaCl for 12 h and then fixed. The treatment experiments were performed in the dark at 22 °C.

### 4.2. Germination Assay and Root Length Measurements

To observe the germination rates and primary root lengths, the seeds were placed in Petri dishes on filter paper that had been soaked with distilled water and 10 mL of a solution of a specific NaCl concentration (0.0 mM, 75 mM, 100 mM, 150 mM and 200 mM NaCl). The experiments were assessed at 72 h. Before germination, the lemma was removed from the seeds to exclude it affecting the germination rates. The seeds were determined to be germinated when the primary root protruded the coleorhiza. Root tip measurements (the distance from the tip of root cap to the hair-root zone) were made using Keyence VHX970F digital microscope (Keyence, Osaka, Japan). Three replicates of 20 seeds each were used for each of NaCl treatments. Each experiment was performed in triplicate.

### 4.3. Sample Preparation and Immunocytochemical Analysis of Cell Wall AGPs

To determine the chemistry of the cell wall, JIM8 and JIM13 monoclonal antibodies (Plant Probes, Leeds, UK) against the specific cell wall epitopes of the AGP were used. JIM8 and JMI13 targets the arabinogalactan and (β)GlcA1->3(α)GalA1->2Rha AGP epitopes, respectively [78,79,80]. The roots were excised, fixed and embedded in Steedman’s wax according the procedure described in detail in [81,82]. Slides with transverse sections of the roots were prepared according to Sala et al. [82]. The slides were stained with 0.01% (*w/v*) fluorescent brightener 28 (FB) (Sigma-Aldrich, St. Louis, MO, USA) in phosphate buffered saline (PBS), which was used to visualise cell walls.

### 4.4. EdU Detection

Seedlings were placed on filter paper that had been soaked with a 10 mM EdU and a respective NaCl solution for the final 12 h of growth before fixation in ethanol:glacial acetic-acid (3:1, *v*/*v*). To prepare the slides, the material was washed with a 0.01 M sodium citrate buffer (pH 4.8) for 30 min and digested with 2% cellulase (*w*/*v*, Onozuka, Serva, Heidelberg, Germany) and 20% pectinase (*v/v*, Sigma-Aldrich, Saint Louis, MO, USA) for 2 h at 37 °C. After digestion, the material was washed with a sodium citrate buffer for 30 min. Squash preparations were made in a drop of 45% acetic acid. After freezing and removing the coverslips, the slides were dried. Prior to the EdU detection, the slides were permeabilized with 0.5% Triton X-100 for 15 min and then washed in PBS at room temperature (RT). The slides were incubated for 30 min at RT in an EdU reaction cocktail (Click-iT EdU Imaging Kits Alexa Fluor 488, Invitrogen, Carlsbad, CA, USA), which was prepared according to the manufacturer’s instructions. The slides were mounted and counterstained in Vectashield (Vector, Burlingame, CA, USA) containing 2 µg/mL of DAPI (Sigma-Aldrich). The preparations were examined using a Zeiss Axio Imager.Z.2 wide-field fluorescence microscope (Zeiss, Oberkochen, Germany) equipped with an AxioCam monochromatic camera (Zeiss). Images were captured and processed using Zen 3.1 (Zeiss) based on which the frequency of the EdU positive nuclei (with Alexa Fluor 488 signal) and MI were calculated. For each experimental group (control, salt stress and salt shock) and specific salt concentration, three plants were studied and at least 4000 nuclei were evaluated in per plant.

### 4.5. Nuclei Isolation

Interphase nuclei from the roots were isolated according to the method described by Lysak et al. [83]. At least 50 salt-shocked seedlings from each NaCl concentration and the control were fixed in 4% formaldehyde in 1 × PBS (pH 7.3) at 4 °C for 1 h after fixation, the seedlings were washed twice in an ice-cold 1 × PBS buffer for 10 min. The roots were separated and washed in a Tris buffer (pH 7.5) at 4 °C for 15 min and chopped with a razor blade in a 100 μL LB-01 buffer on ice in a Petri dish. The suspension was filtered through nylon mesh (30 μm pores) and 15 μL of the nuclei suspension were dropped on ice-cold microscopic slides and air-dried. The slides with the isolated nuclei were used for the TUNEL test and for the immunodetection of the modified histones.

### 4.6. TUNEL Test

The TUNEL reaction was used to analyse the DNA damage and was performed on three-day-old seedlings that had been treated with 100 mM or 300 mM NaCl (“salt shock”) and the control. Prior to the TUNEL reaction, the slides were permeabilized by incubating them in 0.1% Triton X-100 (Sigma-Aldrich) in 0.1% sodium citrate at 4 °C for 2 min. The slides were then rinsed with a PBS buffer. Any nuclei with fragmented DNA were detected using the TUNEL reaction mixture (in situ Cell Death Detection Kit, Fluorescein, Roche, Basel, Switzerland). Fifty µL of the TUNEL reaction mixture (enzyme solution—terminal transferase: label solution; 1:9, *v/v*) was applied to each slide followed by incubating them for 1 h at 37 °C in a humid chamber in the dark. The negative control consisted of a reaction mixture without the enzyme. The nuclei were counterstained with DAPI (2 µg/mL) in Vectashield. Images of the nuclei from the different treatments and the control were taken under the same exposure time using the microscope and image acquisition system that was described in Section 4.4. The fluorescence intensities of DAPI and fluorescein were measured as the mean values from the integrated density (IntDen) parameter per nuclei using ImageJ (National Institutes of Health; http://imagej/nig.gov). Integrated density parameter is a sum of all of the pixels within a region of interest (ROI). The eight-bit images with fluorescein and DAPI fluorescence were segmented with the threshold value parameter, their fluorescence intensities were measured as the mean values from the integrated density parameter using ImageJ, and the results of these measurements were estimated in the relative units. An average of 400 nuclei were analysed for each experimental group.

### 4.7. Chromatin Immunostaining

To detect epigenetic modifications three-day-old seedlings that had been treated with 100 and 300 mM NaCl (“salt shock”) and control were used. The following rabbit monoclonal and polyclonal antibodies against the selected modification of histones H3 and H4 were used: H3K4me3 (Merck-Millipore, Burlington, MA, USA, cat. no. 07-473), H3K27me3 (Merck-Millipore, cat. no. 07-449) and H4K5ac (Merck-Millipore, cat. no. 04-118). As the secondary antibody, Alexa Fluor 488 goat anti-rabbit IgG (Thermo Fisher Scientific, Waltham, MA, USA, cat. no. A-11008) was used. The antibodies were diluted 1:100 in 1% BSA in 1% PBS. Immunostaining was performed as was described in [84]. The cytosine methylation was detected using mouse antiserum raised against 5-methylcytosine (1:500 dilution in 1% BSA in 1 × PBS; Abcam, Cambridge, UK, cat. no. ab10805) and Alexa 488 goat anti-mouse secondary antibody (Thermo Fisher Scientific, cat. no. A-11001). The DNA of the nuclei was denatured using a mixture of 0.5 M NaOH and 1 M NaCl for 30 min at RT. Then, the slides were washed four times for 5 min in distilled water and neutralized in 0.4 M Tris-HCl for 30 min at RT. The denatured DNA was dehydrated in an ethanol series (70%, 90%, 100%; 5 min each), the slides were air-dried and then blocked in 5% BSA for 1 h at RT. The slides were incubated with the primary antibody overnight at 4 °C. After washes in 1 × PBS, the secondary antibody was applied. Nuclei were counterstained as was described in Section 4.4. The quantitative acquisition and analysis were performed using a high-content ScanˆR screening system (Olympus, Tokyo, Japan), which was based on a wide-field Olympus IX81 microscope equipped with a CCD ORCA-ER camera (Hamamatsu Photonics, Hamamatsu, Japan) and an MT20 illumination system, which was based on a 150-W xenon mercury lamp. The analysis was performed as was described in Braszewska-Zalewska et al. [85]. Approximately 2000 nuclei for each modification and each NaCl concentration and the control were analysed. The total number of nuclei from each experimental group was divided into two groups containing 2 C DNA (G1) and 4 C DNA (G2) based on the mean fluorescence intensity of the DAPI. The fluorescence of Alexa 488 was measured for nuclei from the G1 and G2 phases and is presented on the graphs. The G1 and G2 phases of the nuclei were segmented using total DAPI fluorescence intensity into two population of nuclei with 2 C and 4 C DNA content. The significant differences in Alexa 488 fluorescence between the salt-treated plants and the control were calculated using the Student’s test.

### 4.8. RT-qPCR

RT-qPCR was performed using a LightCycler^®^ 480 SYBR Green I Master in a LightCycler^®^ 480 Real-Time PCR System (Roche). The total RNA was isolated from the “salt stressed” and “salt shocked” Brachypodium roots using a NucleoSpin^®^ RNA Plant and Fungi (Macherey-Nagel, Dueren, Germany) commercial kit and then treated with DNase (QIAGEN, Hilden, Germany); both steps were performed according to the manufacturers’ protocol. Subsequently, isolated RNA was used to generate the first-strand cDNA. For the reverse transcription, the PCR conditions were as follows: 5 min at 95 °C, 45 cycles of 10 s at 95 °C, 20 s at 60 °C and 10 s at 72 °C with signal acquisition. The gene encoding ubiquitin, which serves as routine benchmark to normalize tested genes, was used as the internal control. The expression levels for all of the genes in three biological replicates were calculated and normalized using the 2^−ΔΔC_T_^ method [86]. The significant differences between the samples and the control were calculated using the Student’s *t*-test (*p* < 0.05). The sequences of the primers that were used are listed in Table 1.

### 4.9. Metabolomics Sample Preparation, Extraction and Data Analysis

For the metabolomic analysis of the glutathione accumulation, the metabolites from the frozen roots of the salt-treated Brachypodium (40 mg) were extracted using a single-phase extraction solution (chloroform:methanol:water, 1:2.5:1, *v/v/v*). For this purpose, the frozen roots were homogenized and mixed with 1 mL of the extraction solution for 20 min at 4 °C. Next, the samples were centrifuged for 30 min at 4 °C and the supernatant was transferred into new tubes from which 200 μL were taken for further analyses. In the meantime, the master mix was prepared from 20 μL of each sample and a pure extraction solution was used as the blank. Metabolite fingerprinting was performed using high-resolution flow infusion electrospray ionization—MS (FIE—HRMS) using a Q executive plus mass analyzer instrument with a UHPLC system (Thermo Fisher Scientific). The m/z were generated in both the positive and negative ionization modes in a single run as was described by Baptista et al. [87]. Metabolomic analyses were performed using MetaboAnalyst 4.0 (https://www.metaboanalyst.ca/). The significance of the cross-validated *p*-values, based on the one-way analysis of variance (ANOVA) was set to *p* < 0.05. The multiple comparison and post hoc analysis used Tukey’s honestly significant difference (Tukey’s HSD). For each mass, -ion (*m*/*z*) annotation was made using a 5-ppm tolerance on their accurate mass. A metabolic set enrichment analysis using the Mummichog programme within MetaboAnalyst was performed to identify any biologically meaningful patterns.

## 5. Conclusions

In this work, we provide some molecular landmarks that change and likely influence the negative response to salt treatment during germination in Brachypodium. We demonstrate that a 100 mM concentration is the threshold NaCl dose that enables the mitotic activity and DNA replication events. We suggest that increased AGP expression in response to salt, mostly likely linked to cell wall localized events, is associated with altered growth patterns. The inhibition of the cell cycle activity and gene expression in the plants that had been exposed to higher concentrations of NaCl may have been triggered by the toxic effect of Na^+^ ion accumulation. In general, we conclude that salinity stress inhibits seed germination and root growth, causes changes in the histone modification, disrupts the cell cycle and alters the expression of key cell cycle- and cell wall-associated genes. Our observed landmarks will facilitate the design of follow-on experiments which will deepen our knowledge of plant responses to salinity stress. Given our focus on Brachypodium it is likely that these will be relevant for plant breeding programs aiming to develop salt-tolerant crop cultivars.

## Figures and Tables

**Table 1:** The oligonucleotide primers that were used in RT-qPCR reaction.

Gene Name	Gene Description	Primer Sequence (5′—3′)
*AK437296*	Ubiquitin	pF-TCAAAATGCAAGAACGCAAA pR-TCCACACTCCACTTGGTGCT
*Bradi3g02270.1*	cyclin-dependent kinase (CDK), subfamily CDKA	pF-CGAGAAGGTGGAGAAGATCG pR-CGATGGTCTCGTTGGTGTAG
*Bradi4g25980.1*	cyclin-dependent kinase (CDK), subfamily CDKB1	pF-AAGTGTACAAGGCGCAGGAC pR-ATCCCTTCGTCGTCCATCTC
*Bradi3g40200.1*	cyclin-dependent kinase (CDK), subfamily CDKB2	pF-AGGGCCAGACCATCCTCTAC pR-GGATCTTCTCGTGGTTCTGG
*Bradi1g14820.1*	CYCLIN, subfamily CYCA3	pF-ATCCTTGTTGACTGGCTCGT pR-CGGTCGATGTAGGAGATGGT
*Bradi2g52760.1*	CYCLIN, subfamily CYCB1	pF-GTCCTGGGAAAGCAGAAGGT pR-GGACGTTGACGACGTTGC
*Bradi4g32556.1*	CYCLIN-D4-RELATED	pF-CTTGTCTGTAGCGGCCAAGA pR-CTGGATCGTCATGGCTTCGA
*Bradi3g03112.3*	wee1-like protein kinase (WEE1)	pF-AGGATTTCTTCTGCACCCCG pR-GGAGATTTGGGGCAAGGGAT
*Bradi2g00220*	fasciclin-like arabinogalactan protein	pF-AGCTCAACAGCTCCCAGAC pR-CGAAAGCGAGTTGAGCGTG
*Bradi5g18950*	fasciclin-like arabinogalactan protein	pF-AATAAAGGGAAGTCACCGTCGC pR-CCGTTCTTCTTGTCATGGACCT
*Bradi4g34420*	fasciclin-like arabinogalactan protein	pF-CACATCCTCCAGATGCACGTC pR-CCGGACTCCTGGAACATGG
*Bradi2g45510*	arabinogalactan protein	pF-GTGCCCGTGTACTACTCTC pR-CGTGAAGTTGAAGTTCTTG
*Bradi2g60270*	arabinogalactan protein	pF-AGCAGAGCAATCCTCTAGTAGC pR-TGGGTTCTTCTCGCCATTGTTA

## Data Availability

The data presented in this study are available on request from the corresponding authors. The data are not publicly available as they are contained in laboratory notebooks.

## Supplementary Materials

Supplementary Materials can be found at https://www.mdpi.com/1422-0067/22/2/949/s1. Figure S1. The mitotic activity of the root cells in the Brachypodium seedlings that had been “salt-stressed” (**A**) and “salt-shocked” (**B**) with various concentrations of NaCl and the frequency of nuclei with replicated DNA in the root meristematic cells.
