# Supplementary material for: Defining the Cell Wall, Cell Cycle and Chromatin Landmarks in the Responses of Brachypodium distachyon to Salinity
Source: Int J Mol Sci. 2021 Jan 19;22(2):949. doi: 10.3390/ijms22020949 (PMC7835837; doi:10.3390/ijms22020949)
Supplement: Supplementary file 1 [file ijms-22-00949-s001.pdf]

International Journal of
Molecular Sciences

MDPI

Supplementary Materials

Article Title: Defining the Cell Wall, Cell Cycle and Chromatin Landmarks in the Responses of *Brachypodium Distachyon* to Salinity

Authors: Elzbieta Wolny, Aleksandra Skalska, Agnieszka Braszewska-Zalewska, Luis A. J. Mur and Robert Hasterok

The following Supplementary Materials are available for this article:

Supplementary Figure

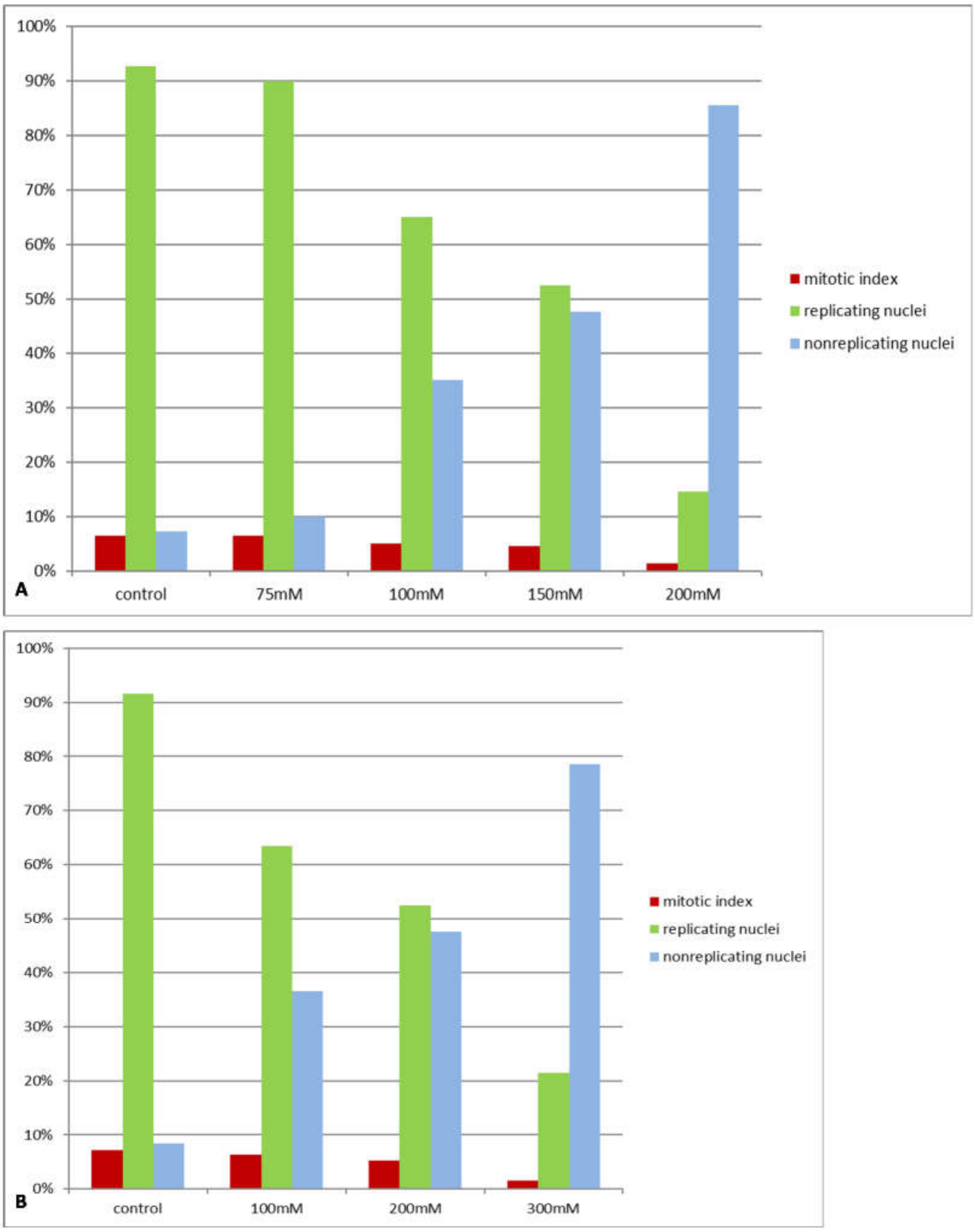

Figure S1. The mitotic activity of the root cells in the Brachypodium seedlings that had been "salt-stressed' (**A**) and "salt-shocked" (**B**) with various concentrations of NaCl and the frequency of nuclei with replicated DNA in the root meristematic cells.
